# Efficacy and Safety of Topical and Systemic Therapies for Atopic Dermatitis in Patients With Skin of Colour: A Systematic Review

**DOI:** 10.7759/cureus.115727

**Published:** 2026-09-03

**Authors:** Muhammad Waqas, Inzamam Sarwar, Hirra Junejo, Zohaib Ansari, Ben Alexander Marshall

**Affiliations:** 1 General Medicine, Cumberland Infirmary, Carlisle, GBR; 2 General Practice, District Headquarters (DHQ) Allied Hospital 2, Faisalabad, PAK; 3 Internal Medicine, North Cumbria Integrated Care NHS Foundation Trust, Cumbria, GBR; 4 Acute Medicine, University of Aberdeen, Carlisle, GBR

**Keywords:** atopic dermatitis, dupilumab, eczema, ethnicity, fitzpatrick skin type, race, skin of colour, treatment efficacy

## Abstract

Atopic dermatitis (AD) is more common and often more severe in people with skin of colour, yet the trials that shaped modern treatment enrolled mostly White participants. The disease can look different in darker skin, and erythema-based severity scores may miss active inflammation. Whether current topical and systemic drugs work as well, and are as safe, across racial, ethnic, and Fitzpatrick skin-type groups, is still uncertain. This review brought together the evidence on AD treatment outcomes reported by skin colour. Preferred Reporting Items for Systematic reviews and Meta-Analyses (PRISMA) 2020 and the Synthesis Without Meta-analysis (SWiM) guidance were followed, and PubMed and Scopus were searched from 2015 to March 2026. Randomised controlled trials, their pre-specified or post hoc subgroup analyses, and prospective cohort or registry studies were included if they reported efficacy or safety of any topical or systemic AD drug either stratified by race, ethnicity, or Fitzpatrick skin type, or in a dedicated skin-of-colour population. Two reviewers independently screened studies and extracted data. A meta-analysis was not feasible due to substantial heterogeneity in study designs, interventions, outcome measures, and reporting methods, as well as insufficient quantitative data for statistical pooling. Fifteen studies met the criteria, covering topical agents (tapinarof, crisaborole, roflumilast), interleukin-targeted biologics (dupilumab, tralokinumab, lebrikizumab), Janus kinase inhibitors (abrocitinib, upadacitinib, baricitinib), and conventional systemics in a registry cohort. Across drug classes, response rates among patients with skin of colour were clinically meaningful and broadly consistent with those in lighter-skin strata. Tapinarof reached the primary endpoint in Asian, Black or African American, and White patients and across Fitzpatrick I-III and IV-VI groups. Tralokinumab achieved Eczema Area and Severity Index (EASI-75) rates of 78%, 88%, and 83% in Asian, Black, and White patients after up to two years. Signals of possible difference were small and imprecise, mostly in Black or African American subgroups, which were consistently the smallest, and safety profiles matched those in the wider trial populations. A recurring limitation was that erythema-based tools may underestimate severity in dark skin. Topical and systemic AD therapies are effective and well tolerated in patients with skin of colour, with no clear evidence of reduced benefit. The evidence is limited by small, often untested subgroups, especially Black or African American and Fitzpatrick V-VI patients, and by measurement tools that were not built for darker skin. Dedicated trials and skin-tone-valid outcome measures are needed.

## Introduction and background

Atopic dermatitis (AD) affects people of every ancestry, but the burden is not shared equally. Black and Asian populations carry a higher prevalence than White populations in many countries, and Black children in particular tend to present with more severe and more persistent disease [1,2]. The way AD looks on darker skin can also differ. Instead of the bright erythematous plaques described in textbooks, patients with skin of colour more often show follicular, papular, or lichenoid lesions, a violaceous rather than red colour, and prominent post-inflammatory hyperpigmentation or hypopigmentation [3-5]. These pigmentary changes can outlast the inflammation and add their own weight to the quality of life.

The difference in appearance creates a measurement problem. Most validated severity tools, including the Eczema Area and Severity Index (EASI) and scoring systems based on erythema, were designed and tested primarily in lighter skin. Redness is harder to detect against a melanin-rich background, so erythema-based grading can understate the disease's activity in darker skin [6]. A patient with Fitzpatrick type V or VI skin may therefore be scored as milder than they are, which affects both trial enrolment and the reading of trial results. The Fitzpatrick skin type is a widely used clinical scale that grades skin by its constitutive colour and its response to sun exposure, with types I to III denoting lighter skin and types IV to VI denoting darker skin.

Underlying all of this is a representation gap. A recent analysis of 68 AD trials with more than 20,000 participants found that 65.4% were White, 17.3% Asian, and 13.4% Black or African American, while Hispanic or Latino patients made up only 10.8% and were the most under-counted [7]. Because the main registration trials enrolled few patients of colour, and rarely broke their results down by skin colour, clinicians have had little direct evidence to answer a simple question: does this drug work as well in my patient with darker skin?

Earlier attempts to synthesise this area left clear gaps. An older review looked at systemic therapy in United States racial and ethnic minorities but predated the biologic and Janus kinase (JAK) inhibitor era [8]. The most relevant recent synthesis, by Rijal and colleagues, examined biologic therapy in skin-of-colour participants but combined AD with psoriasis, was restricted to biologics, and found too few data to pool [9]. None of these covered the newer topical agents such as tapinarof and roflumilast, and none captured the 2025 and March 2026 analyses in which phase 3 programmes finally reported efficacy by race and Fitzpatrick skin type.

This review was designed to fill that space. The aim was to synthesise the efficacy and safety of both topical and systemic AD therapies in patients with skin of colour across all major drug classes, drawing on studies that either stratified outcomes by race, ethnicity, or Fitzpatrick skin type, or were conducted in dedicated skin-of-colour populations. Four features separate this work from what came before. It includes topical anti-inflammatory therapies, not biologics alone. It helps distinguish AD from psoriasis. It asks about treatment response and safety rather than trial enrolment, and it incorporates the most recent skin-of-colour datasets, including the tapinarof ADORING stratified analyses [10] and the first dedicated skin-of-colour biologic trial, which earlier reviews could not have seen. Establishing whether treatment response and safety differ by skin colour has direct clinical relevance, as it informs whether the same evidence-based topical and systemic therapies can be offered at the same thresholds to patients across the full range of skin tones.

## Review

Methodology

Protocol

This systematic review was conducted in accordance with the Preferred Reporting Items for Systematic Reviews and Meta-Analyses (PRISMA) 2020 guidelines [11]. A meta-analysis was not feasible because the included studies differed substantially in drug classes, study designs, outcome measures, and reporting methods, so the findings were synthesised and reported in accordance with the SWiM (Synthesis Without Meta-analysis) reporting guidelines [12].

Eligibility Criteria

The eligibility criteria were built around a Population, Intervention, Comparison, and Outcome (PICO) framework. The population was patients of any age with a clinical diagnosis of AD who had skin of colour, which was taken to mean non-White race or ethnicity (Black or African American, Asian, Hispanic or Latino, and related groups) or Fitzpatrick skin phototypes IV to VI. Two routes into the review were allowed. A study was considered eligible if it reported outcomes for a skin-of-colour subgroup within a broader trial, and those data could be extracted separately. A study also qualified if it was carried out in a dedicated skin-of-colour population, such as a trial run entirely in Chinese or Japanese patients, because that population is itself a skin-of-colour group and the result is a skin-of-colour result.

The intervention was any topical or systemic pharmacological therapy for AD. This covered topical agents (tapinarof, crisaborole, roflumilast, topical corticosteroids, topical calcineurin inhibitors, and ruxolitinib); systemic agents (dupilumab, tralokinumab, lebrikizumab, and the JAK inhibitors abrocitinib, upadacitinib, and baricitinib); and conventional systemics such as methotrexate and ciclosporin. Comparators could be a placebo, a vehicle, an active drug, standard care, or none, since single-arm subgroup reporting was eligible.

The primary outcomes were efficacy measures reported by skin colour: the proportion of patients reaching an Investigator's Global Assessment (IGA) or validated IGA for Atopic Dermatitis (vIGA-AD) of clear or almost clear [10,13,14], EASI-75, and changes in EASI [15,16], SCORing Atopic Dermatitis (SCORAD) [13,17], and peak pruritus scores [13,18]. Secondary outcomes were safety and tolerability, including treatment-emergent adverse events, serious adverse events, and discontinuations in the skin-of-colour population, as well as any direct comparisons of efficacy or safety between skin-of-colour and lighter-skin strata.

The primary evidence base comprised randomised controlled trials, controlled clinical trials, and their pre-specified or post hoc subgroup analyses. Prospective observational and registry cohorts that reported outcomes by skin type were eligible as supporting evidence. The following were excluded: studies confined to White or Fitzpatrick I-III patients with no separate skin-of-colour data, studies that did not break outcomes down by race, ethnicity, or skin type and were not run in a dedicated skin-of-colour population, non-AD skin conditions, conference abstracts, editorials, letters, narrative reviews without original data, retrospective real-world case series, non-English publications, animal or preclinical work, pharmacokinetic-only reports, and duplicates. Studies of enrolment representation or treatment access that carried no treatment-efficacy outcome were also excluded, though they were drawn on for context.

Information Sources and Search Strategy

PubMed and Scopus were the main databases. Both final searches ran on 1st March 2026 and covered publications from January 1, 2015 onward. The search was limited to English-language records. The core PubMed strategy combined three concept blocks with the Boolean operator AND: a disease block ("atopic dermatitis", "atopic eczema", eczema), a skin-colour block ("skin of color" or "skin of colour", Fitzpatrick, "skin type", "skin phototype", race, racial, ethnic, ethnicity, "African American", Black, Asian, Hispanic, Latino), and a treatment or outcome block (treatment, therapy, efficacy, effectiveness, response). The same logic was run in Scopus using TITLE-ABS-KEY fields. A randomised-trial filter was deliberately left out at the search stage, because such a filter would have dropped subgroup analyses that are not tagged as trials.

Study Selection and Data Extraction

Records from both databases were combined in EndNote (Version 21; Clarivate, Philadelphia, PA, USA), and duplicates were removed with the software's duplicate-detection function. Two reviewers screened titles and abstracts against the criteria, then read the full text of every record that passed. Where the two screens disagreed, the reviewers discussed the record and, if needed, a third reviewer made the decision. The reason for each full-text exclusion was recorded.

For every included study, the following were extracted: the study design, country or region, sample size, the exact definition of skin colour used (the race or ethnicity categories and any Fitzpatrick grouping), the intervention and comparator, the outcome measures and their timepoints, and the efficacy and safety results for the skin-of-colour group. Extraction was done in duplicate, and the two versions were reconciled. Study authors were not contacted for unpublished data, so all figures come from the published reports.

Risk of Bias Assessment

Two reviewers independently assessed the risk of bias and resolved differences through discussion. Randomised trials and their subgroup analyses were judged with the Cochrane Risk of Bias 2 (RoB 2) tool [19]. Non-randomised studies, including the prospective registry cohort, were judged with the Risk Of Bias In Non-randomised Studies of Interventions (ROBINS-I) tool [20]. Because several of the efficacy results came from post hoc subgroup analyses that were not powered for the subgroup, particular attention was paid to selective reporting and to imprecision in small strata. Reporting bias was assessed narratively. In keeping with the protocol, certainty was not formally graded with Grading of Recommendations Assessment, Development and Evaluation (GRADE) [21].

Synthesis Methods

The studies split across different drug classes, used different outcome instruments including vIGA-AD, Investigator's Static Global Assessment (ISGA), SCORAD, and EASI, mixed randomised subgroup data with registry cohorts, and often reported subgroup response rates without dispersion data or formal between-group testing. Pooling numbers of that kind would have produced a falsely precise answer. Following SWiM, studies were grouped in two ways: by drug class (topical versus systemic or biologic versus JAK inhibitor) and by skin-colour stratum (race or ethnicity category, and Fitzpatrick I-III versus IV-VI). The effect estimates were tabulated as reported in the primary studies and, where a formal test was absent, summarised the consistency of findings by effect direction. Sources of heterogeneity, above all the validity of erythema-based outcome measures in darker skin, were explored in words rather than statistics. Where the primary studies reported them, effect estimates are presented with their 95% confidence intervals, and within-subgroup treatment effects are kept separate from formal between-subgroup interaction tests. Because most subgroup analyses were not powered for the subgroup and few reported interaction testing, the findings are described by the direction and consistency of effect across strata rather than as formal statistical equivalence between skin-colour groups. The absence of a meta-analysis reflects the heterogeneity and limited quantitative reporting in the primary studies, and it should not be read as an absence of a treatment effect.

Results

Study Selection

The two database searches returned 1,825 records, 435 from PubMed and 1,390 from Scopus. After EndNote (Version 21; Clarivate, Philadelphia, PA, USA) removed 341 duplicates, 1,484 records went forward to title and abstract screening. Most were off-topic. A large share were comorbidity or epidemiology records that mentioned eczema only in passing (e.g., studies of dementia, otitis media, or keratoconus risk), and these were removed quickly. Screening left 96 reports for full-text assessment. After full-text assessment, we excluded 81 reports: 29 did not stratify outcomes by race, ethnicity, or skin type and were not run in a dedicated skin-of-colour population; 24 were retrospective real-world cohorts or case series outside the prospective supporting-evidence rule; nine were case reports; 11 were conference abstracts, reviews, or reports with no original data; five were representation or access studies with no treatment-efficacy outcome; and three involved a non-AD condition or an ineligible intervention such as a pharmacokinetic-only study. Fifteen studies met all criteria and were included in the synthesis. The selection process is shown in Figure 1.

**Figure 1:**
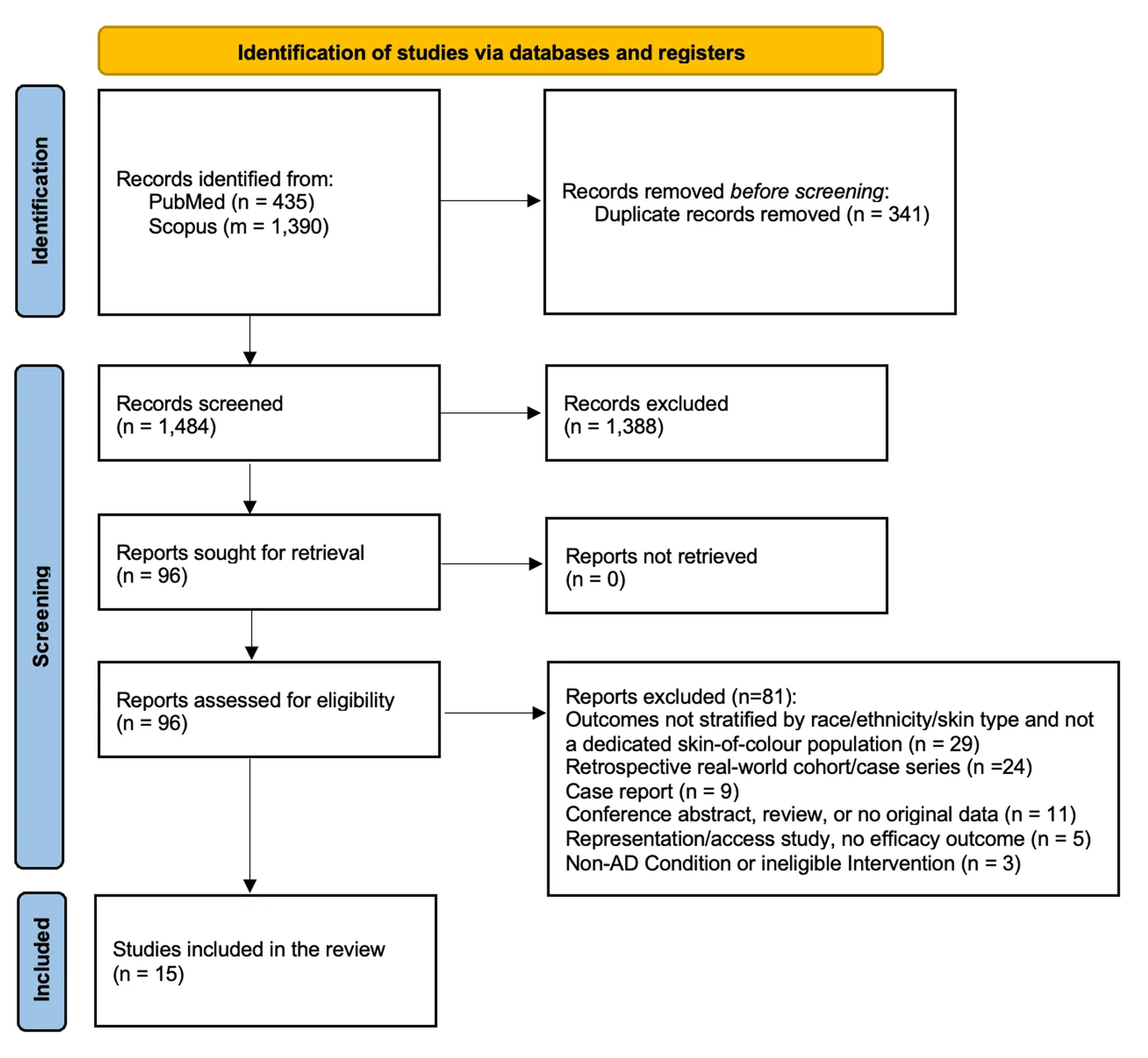
PRISMA flow diagram of the study selection process PRISMA: Preferred Reporting Items for Systematic Reviews and Meta-Analyses; AD: atopic dermatitis

Characteristics of Included Studies

They fell into two evidence streams. The first stream, comprising nine studies, reported efficacy or safety stratified by race, ethnicity, or Fitzpatrick stratum within multi-country trial programmes. A second stream of six studies was conducted in dedicated skin-of-colour populations, mostly East Asian registration trials, plus one prospective European registry that directly compared dark and light skin types. Race or ethnicity, Fitzpatrick skin phototype, and dedicated geographic populations are related but distinct ways of defining skin colour, and the evidence is reported here as separate streams rather than pooled, so that these constructs are not treated as interchangeable.

By drug class, three studies covered topical agents: tapinarof [10,15], crisaborole [14,22], and roflumilast [23]. Six studies covered interleukin-targeted biologics: dupilumab [17,18,24,25], tralokinumab [26], and lebrikizumab [16,27]. Four studies covered JAK inhibitors: abrocitinib [13], upadacitinib [28], and baricitinib [29]. The Bosma registry also captured methotrexate and ciclosporin alongside dupilumab [25]. Sample sizes ranged from 90 in the dedicated skin-of-colour lebrikizumab trial [27] to more than 2,500 in the pooled analyses of upadacitinib and dupilumab [28,29]. Most efficacy studies used vIGA-AD or IGA 0/1 and EASI-75 as their main endpoints, with peak pruritus and SCORAD reported in several. The 15 studies are presented in Table 1.

**Table 1:** Characteristics of the 15 included studies AD: Atopic dermatitis; AhR: aryl hydrocarbon receptor; DLQI: Dermatology Life Quality Index; EASI: Eczema Area and Severity Index; FST: Fitzpatrick skin type; GISS: Graphic Investigator's Static Score; IGA/vIGA-AD: (Validated) Investigator Global Assessment (for atopic dermatitis); IL-4Rα: interleukin-4 receptor alpha; IL-13: interleukin-13; ISGA: Investigator's Static Global Assessment; PDE4: phosphodiesterase-4; PDCA-Derm: Post-Inflammatory Dyspigmentation Clinical Assessment for Dermatology; POEM: Patient-Oriented Eczema Measure; PP-NRS: Peak Pruritus Numerical Rating Scale; QoL: Quality of Life; RCT: randomised controlled trial; SCORAD: Scoring Atopic Dermatitis; TEAE: treatment-emergent adverse event; WI-NRS: Worst Itch Numerical Rating Scale

Study	Design; n	Skin-of-colour population/strata	Intervention (class)	Outcome measures
Alexis et al. 2025 [10]	Post hoc stratified analysis, 2 phase 3 RCTs (ADORING 1 & 2); n=813	Asian, Black/African American, White; Fitzpatrick I–III vs IV–VI (>50% IV–VI)	Tapinarof 1% cream (topical AhR agonist)	vIGA-AD 0/1; EASI-75
Callender et al. 2019 [14]	Pooled post hoc, 2 phase 3 RCTs + safety extension	White vs nonwhite; Hispanic/Latino vs not	Crisaborole 2% (topical PDE4)	ISGA success; signs/symptoms; QoL
Prajapati et al. 2026 [23]	Subgroup analysis, phase 3 RCT (INTEGUMENT-PED); n=651; age 2–5 y	White/non-White; Hispanic; Fitzpatrick I–III vs IV–VI	Roflumilast 0.05% cream (topical PDE4)	vIGA-AD success; EASI-75; WI-NRS
Alexis et al. 2019 [18]	Pooled post hoc, 3 phase 3 RCTs (SOLO 1/2, CHRONOS); n=2058	White (1429), Asian (501), Black/African American (128)	Dupilumab (IL-4Rα)	EASI; peak pruritus NRS; IGA; DLQI; POEM
Guttman-Yassky et al. 2025 [17]	Post hoc pooled, 5 RCTs; n=1902 (by race)	Asian, Black/African American, White	Dupilumab (IL-4Rα)	Lichenification (GISS); SCORAD; EASI
Mayo et al. 2026 [26]	Post hoc, 4 phase 3 trials (ECZTRA 1/2/3 + ECZTEND); n=1876	Asian (407), Black (134), White (1335)	Tralokinumab (IL-13)	IGA 0/1; EASI-75; adverse events; biomarkers
Alexis et al. 2025 [27]	Open-label phase 3b, dedicated skin of colour (ADmirable); n=90	Non-White race; Fitzpatrick IV (43%), V (24%), VI (32%)	Lebrikizumab (IL-13)	EASI-75/90; IGA 0/1; pruritus; pigment (PDCA-Derm)
Alexis et al. 2024 [13]	Pooled post hoc, 3 monotherapy trials; n=915	White (628), Asian (204), Black (83); FST I–III/IV–VI	Abrocitinib (JAK1)	IGA 0/1; EASI-75; SCORAD-75; PP-NRS; DLQI; POEM
Thyssen et al. 2023 [28]	Post hoc stratified analysis, 3 phase 3 RCTs; n=2584	Race among baseline subgroups	Upadacitinib (JAK1)	EASI-75; vIGA-AD 0/1; itch
Ma et al. 2023 [22]	Phase 3 RCT (CrisADe CLEAR); age ≥2 y	Dedicated East Asian (Chinese, Japanese)	Crisaborole 2% (topical PDE4)	EASI %change; ISGA; PP-NRS
Igarashi et al. 2024 [15]	2 Japanese phase 3 trials (ZBB4-1 RCT, ZBB4-2 open-label); n=507	Dedicated Japanese	Tapinarof 1% cream (topical AhR agonist)	IGA success; EASI-75
Katoh et al. 2020 [24]	Subanalysis of Japanese cohorts, 3 trials; n=250	Dedicated Japanese	Dupilumab (IL-4Rα)	Signs/symptoms; pruritus; QoL; safety
Katoh et al. 2025 [16]	Phase 3 RCT (ADhere-J) + topical corticosteroids; n=286	Dedicated Japanese	Lebrikizumab (IL-13)	IGA 0/1; EASI-75; safety
Katoh et al. 2022 [29]	Pooled safety, 6 RCTs, Japanese subpopulation; n=341	Dedicated Japanese	Baricitinib (JAK1/2)	TEAEs; adverse events of special interest
Bosma et al. 2023 [25]	Prospective registry cohort (TREAT NL + A-STAR); n=235	Light (FST 1–3, n=156) vs dark (FST 4–6, n=68)	Dupilumab, methotrexate, ciclosporin	EASI change; adverse events

Race and ethnicity were self-reported in every stratified analysis, and Fitzpatrick skin type was investigator-assessed. Across the stratified trials, the Black or African American subgroup was consistently the smallest. In the pooled dupilumab analysis, for example, only 128 of 2,058 patients were Black or African American [18], and in the abrocitinib analysis, only 83 of 915 were Black [13]. Asian patients were better represented, in part due to dedicated East Asian trials.

Risk of Bias

The randomised trials themselves were generally at low risk of bias for the core endpoints, since they were double-blind, vehicle- or placebo-controlled, and pre-registered. Most concern sat with the subgroup analyses. Nearly all of the racial and Fitzpatrick comparisons were post hoc and not powered for the subgroup, so results in small strata, especially among Black or African American patients, carried a real risk of imprecision, and several reports reported response rates without confidence intervals or formal interaction tests. The open-label, single-arm design of the dedicated lebrikizumab skin-of-colour trial reduced its certainty [27], and it was assessed using ROBINS-I as having a moderate risk owing to the absence of a control arm. In the prospective registry [25], which was unblinded and non-randomised, confounding by indication was the main threat, and it was rated as a moderate-to-serious risk on the ROBINS-I. No study was excluded on the basis of risk of bias alone. The risk-of-bias judgements are given in Table 2.

**Table 2:** Risk-of-bias assessment (Cochrane RoB 2 and ROBINS-I) AA: African American; RoB 2: Risk of Bias 2; ROBINS-I: Risk of Bias in Non-randomised Studies of Interventions; ZBB4-1: Zirconia Broken Barrier 4-1 trial; ZBB4-2: Zirconia Broken Barrier 4-2 trial

Study	Tool	Overall risk	Main concern
Alexis et al. 2025 [10]	RoB 2	Low to some concerns	Post hoc subgroup analysis
Callender et al. 2019 [14]	RoB 2	Some concerns	Post hoc; broad nonwhite grouping
Prajapati et al. 2026 [23]	RoB 2	Low to some concerns	Subgroup analysis
Alexis et al. 2019 [18]	RoB 2	Some concerns	Small Black/AA subgroup; imprecision
Guttman-Yassky et al. 2025 [17]	RoB 2	Some concerns	Post hoc; single sign (lichenification)
Mayo et al. 2026 [26]	RoB 2	Low to some concerns	Post hoc; open-label extension
Alexis et al. 2025 [27]	ROBINS-I	Moderate	Single-arm, open-label, no control
Alexis et al. 2024 [13]	RoB 2	Some concerns	Small Black subgroup
Thyssen et al. 2023 [28]	RoB 2	Low to some concerns	Race-specific rates not reported
Ma et al. 2023 [22]	RoB 2	Low	None noted
Igarashi et al. 2024 [15]	RoB 2	Low (ZBB4-1); moderate (ZBB4-2)	ZBB4-2 open-label, uncontrolled
Katoh et al. 2020 [24]	RoB 2	Low to some concerns	Subanalysis
Katoh et al. 2025 [16]	RoB 2	Low	None noted
Katoh et al. 2022 [29]	RoB 2	Low to some concerns	Pooled safety only
Bosma et al. 2023 [25]	ROBINS-I	Moderate to serious	Confounding by indication; unblinded

Efficacy of Topical Therapies

The topical evidence was reassuring and, for tapinarof and roflumilast, included a genuine spread across skin types. In the ADORING 1 and 2 phase 3 trials, more than half of the 813 randomised patients had Fitzpatrick skin types IV to VI, and tapinarof cream 1% once daily met the primary vIGA-AD endpoint at week 8 across all racial groups [10]. Response rates were consistently higher with tapinarof than vehicle across Asian, Black or African American, and White patients, and across Fitzpatrick I to III and IV to VI groups. EASI-75 followed the same pattern; detailed figures are given in Table 3. The Japanese phase 3 programme supported this, with tapinarof reaching EASI-75 in 40.3% of patients versus 4.3% on vehicle at week 8 and 76.6% by week 52 in the open-label trial [15].

**Table 3:** Key efficacy findings by drug class and skin-of-colour stratum AA: African American; DLQI: Dermatology Life Quality Index; EASI: Eczema Area and Severity Index; EASI-75: ≥75% improvement in Eczema Area and Severity Index; FST: Fitzpatrick skin type; IGA: Investigator's Global Assessment; IL-4Rα: interleukin-4 receptor alpha; IL-13: interleukin-13; ISGA: Investigator's Static Global Assessment; JAK1: Janus kinase 1; RCT: randomized controlled trial; SoC: skin of colour; vIGA-AD: Validated Investigator Global Assessment for Atopic Dermatitis Values are the proportions reported in each primary study; formal between-group tests were often not performed.

Drug (class)	Skin-of-colour strata	Key efficacy finding
Tapinarof (topical)	Asian, Black/AA, White; Fitzpatrick I–III & IV–VI	Primary vIGA-AD 0/1 met in every group. Tapinarof vs vehicle: Asian 39.5–48.9% vs 3.7–18.5%; Black/AA 43.1–47.0% vs 17.5–24.1%; White 49.4–52.1% vs 12.2–14.5%. Consistent across Fitzpatrick strata [10,15]
Crisaborole (topical)	White/nonwhite; Hispanic; Chinese/Japanese	ISGA success at day 29 higher than vehicle in White (33.5% vs 22.3%), nonwhite (30.0% vs 21.3%) and Hispanic/Latino (35.4% vs 18.2%); confirmed in a dedicated Chinese/Japanese RCT [14,22]
Roflumilast (topical)	White/non-White; Hispanic; FST IV–VI	vIGA-AD success at week 4 higher than vehicle across all strata (non-White 18.9% vs 10.4%; FST IV–VI 23.7% vs 12.1%) in children aged 2–5 y [23]
Dupilumab (IL-4Rα)	White, Asian, Black/AA; Japanese; dark vs light	Significant vs placebo in White and Asian for all outcomes (p<0.0001); Black/AA significant for EASI, pruritus and DLQI. Greater EASI reduction in dark vs light skin (16.7 vs 9.7; adjusted p=0.032) [17,18,24,25]
Tralokinumab (IL-13)	Asian, Black, White	IGA 0/1 and EASI-75 improved vs placebo in all subgroups at week 16; EASI-75 at week 56 reached 78% (Asian), 88% (Black) and 83% (White) [26]
Lebrikizumab (IL-13)	Dedicated SoC (FST IV–VI); Japanese	EASI-75 69.2% at week 16 and 78.4% at week 24; EASI-75 by Fitzpatrick IV/V/VI 62.9%/88.2%/95.5%; hyperpigmentation improved in ~64% of affected patients [16,27]
Abrocitinib (JAK1)	White, Asian, Black; FST I–III/IV–VI	Both doses better than placebo across every race, ethnicity and phototype group; dose-response clearest in White, and similar for the two doses in the small Black subgroup [13]
Upadacitinib (JAK1)	Race among subgroups	EASI-75, vIGA-AD 0/1 and itch response consistently greater than placebo regardless of race [28]

Crisaborole showed a similar, if more modest, benefit across groups. In a pooled analysis of the two registration trials by race and ethnicity, more crisaborole- than vehicle-treated patients reached ISGA clear or almost clear with at least a two-grade improvement at day 29 across White, nonwhite, Hispanic or Latino, and non-Hispanic or Latino groups, with the detailed figures given in Table 3 [14]. The dedicated CrisADe CLEAR trial in Chinese and Japanese patients confirmed that crisaborole was superior to vehicle for change in EASI (p = 0.0002) and ISGA success (p = 0.0078), with no new safety signals [22].

Roflumilast added the newest topical data and, usefully, reported the youngest patients. In a subgroup analysis of the INTEGUMENT-PED trial in children aged 2 to 5 years, roflumilast cream 0.05% beat vehicle for vIGA-AD success at week 4 across White, non-White, Hispanic or Latino, and Fitzpatrick I to III and IV to VI subgroups, with the detailed figures given in Table 3 [23]. Itch relief moved in the same direction across strata.

Efficacy of Biologic Therapies

Dupilumab had the deepest evidence base. The pooled post hoc analysis of SOLO 1, SOLO 2, and CHRONOS included 2,058 patients (1,429 White, 501 Asian, 128 Black or African American) and showed a significant improvement over placebo at week 16 for all key outcomes in the White and Asian subgroups (p < 0.0001) [18]. In the much smaller Black or African American subgroup, dupilumab significantly improved the EASI endpoints and the mean change in peak pruritus and Dermatology Life Quality Index (DLQI) (p < 0.0001), with positive numerical trends for the remaining outcomes that did not always reach significance. A second pooled analysis of five trials found that dupilumab improved lichenification, which is common and stubborn in darker skin, to a similar degree across Asian, Black or African American, and White patients [17]. The dedicated Japanese subanalysis told the same story, with dupilumab improving signs, symptoms, pruritus, and quality of life against placebo, in line with the global population [24].

Tralokinumab, which targets interleukin-13, was studied by racial subgroup across ECZTRA 1, 2, and 3 and the ECZTEND extension, with a pooled cohort of 1,876 patients at the parent stage (Asian 407, Black 134, White 1,335) [26]. At week 16, tralokinumab improved IGA 0/1 and EASI-75 over placebo in every subgroup. With continued treatment, responses increased further, and by week 56 of ECZTEND, EASI-75 was reached by 78% of Asian, 88% of Black, and 83% of White patients, indicating that the long-term response in Black patients was at least as good as in the other groups. Safety was comparable to placebo across subgroups.

Lebrikizumab provided the single most important new piece of evidence: the first phase 3 trial run entirely in patients with skin of colour. The open-label ADmirable study enrolled 90 patients with self-reported non-White race and Fitzpatrick types IV to VI, the majority Black or African American (78%) and split across type IV (43.3%), V (24.4%), and VI (32.2%) [27]. At week 16, 69.2% reached EASI-75, 44.9% reached EASI-90, and 44.9% reached IGA 0/1, and 58.1% reported at least a four-point fall in pruritus. Responses deepened by week 24, when EASI-75 reached 78.4%. The response by Fitzpatrick type at week 24 was striking, with EASI-75 rates of 62.9%, 88.2%, and 95.5% in patients with Fitzpatrick types IV, V, and VI, respectively. Hyperpigmented areas improved in about two-thirds of affected patients on an objective measure of pigmentation, which matters because dyspigmentation is often what patients with skin of colour most want treated. The dedicated Japanese ADhere-J trial supported the class, with lebrikizumab plus topical corticosteroids achieving EASI-75 in 47.2% and 51.2% of the every-four-week and every-two-week groups, respectively, versus 13.4% on placebo [16].

Efficacy of Janus Kinase Inhibitors

The oral JAK inhibitors showed consistent efficacy across skin colour, though the darkest-skin data were again the thinnest. Abrocitinib was analysed by race, ethnicity, and Fitzpatrick type in a pooled set of three monotherapy trials (628 White, 204 Asian, 83 Black; 624 type I-III, 320 type IV-VI) [13]. Both abrocitinib doses beat placebo for IGA clear or almost clear, EASI-75, SCORAD-75, and pruritus response across every racial, ethnic, and phototype group. The dose-response was clearest in White patients, and in Black patients, the two doses performed similarly, a difference the authors linked to the small Black subgroup rather than to a true biological effect. Upadacitinib, analysed across Measure Up 1, Measure Up 2, and AD Up, showed consistently higher rates of EASI-75, vIGA-AD 0/1, and itch relief than placebo, regardless of race and other baseline factors, although race-specific response rates were not reported numerically in that analysis [28]. For baricitinib, the dedicated evidence was a safety analysis rather than an efficacy one [29].

Direct Comparison Across Skin-Type Strata

Only one included study directly compared treatment effectiveness between dark and light skin rather than reporting each stratum separately. In the pooled TREAT NL and A-STAR registries, 235 adults on dupilumab, methotrexate, or ciclosporin were split into light skin types (Fitzpatrick 1-3, n = 156) and dark skin types (Fitzpatrick 4-6, n = 68) [25]. Patients with dark skin were younger, more often had follicular eczema (22.1% versus 2.6%), and had higher baseline EASI scores (20.1 versus 14.9). After adjustment for baseline severity and other covariates, dark skin types showed a greater mean EASI reduction at six months with dupilumab (16.7 versus 9.7; adjusted p = 0.032), while methotrexate and ciclosporin showed no such difference. Adverse events did not differ by skin type across treatments. The finding is hypothesis-generating rather than definitive, given the observational design, but it runs counter to any worry that biologics might work less well in darker skin. The efficacy findings across drug classes and strata are summarised in Table 3.

Safety and Tolerability

No study found a safety signal specific to patients with skin of colour (Table 4). For the topical drugs, adverse events were mostly mild or moderate, and discontinuations were low; for tapinarof, they were lower on the active drug than on vehicle [10,15]. Crisaborole-related adverse events sat at 7.1% to 8.5% in the registration trials [14]. For dupilumab, injection-site reactions and conjunctivitis were more frequent than on placebo, but the overall profile in Asian and Black or African American patients matched the wider population [18,24]. Lebrikizumab produced only mild-to-moderate events in the skin-of-colour trial, with no serious events, no discontinuations, and a single case of conjunctivitis [27]. The most detailed safety data for a JAK inhibitor came from the Japanese baricitinib pooled analysis of six trials (341 patients, 371.7 patient-years), in which serious infections and herpes zoster were low and similar to placebo, treatment-emergent infections such as herpes simplex were more common on baricitinib, and there were no gastrointestinal perforations, tuberculosis, adjudicated cardiovascular events, venous thromboembolism, or deaths [29]. Abrocitinib adverse events were more common in White and Black patients than in Asian patients, without a change in the type of event [13].

**Table 4:** Safety findings in patients of colour AA: African American; IL-4Rα: interleukin-4 receptor alpha; IL-13: interleukin-13; JAK1: Janus kinase 1; JAK1/2: Janus kinase 1/2; RCT: randomized controlled trial; SoC: skin of colour; TB: tuberculosis; VTE: venous thromboembolism No study reported a safety signal specific to patients with skin of colour.

Drug (class)	Safety and tolerability in skin of colour
Tapinarof (topical)	Adverse events mostly mild or moderate; discontinuations low and lower with tapinarof than vehicle; folliculitis, acne and headache reported [10, 15]
Crisaborole (topical)	Treatment-related adverse events 7.1–8.5%, mainly application-site pain; no new signals in the East Asian RCT [14, 22]
Roflumilast (topical)	Low adverse-event rates, ≥98% mild or moderate; local tolerability favourable across skin types [23]
Dupilumab (IL-4Rα)	Injection-site reactions and conjunctivitis more frequent than placebo; profile in Asian and Black/AA patients consistent with the overall population; no adverse-event difference by skin type in the registry [18, 24, 25]
Tralokinumab (IL-13)	Comparable to placebo across racial subgroups; serious adverse events and discontinuations rare [26]
Lebrikizumab (IL-13)	Only mild-to-moderate events in the skin-of-colour trial; none serious or leading to discontinuation; one case of conjunctivitis [27]
Abrocitinib (JAK1)	Treatment-emergent adverse events more common in White and Black than Asian patients, with no change in event type [13]
Baricitinib (JAK1/2)	Japanese pooled safety (341 patients, 371.7 patient-years): serious infections and herpes zoster low and similar to placebo; herpes simplex higher; no tuberculosis, gastrointestinal perforation, adjudicated cardiovascular events, venous thromboembolism or deaths [29]

Discussion

Summary of Main Findings

The evidence gathered here points in one direction. Across topical agents, interleukin-targeted biologics, and JAK inhibitors, patients with skin of colour treated for AD achieved clinically meaningful responses broadly in line with those observed in lighter-skinned patients. Tapinarof met its primary endpoint in Asian, Black or African American, and White patients and across Fitzpatrick I-III and IV-VI groups [10]. Dupilumab, tralokinumab, abrocitinib, and upadacitinib all beat their comparators across racial strata [13,18,26,28]. The first phase 3 trial, conducted entirely in patients with skin of colour, showed that lebrikizumab cleared or nearly cleared most patients and, in an unusual and welcome step, improved their hyperpigmentation [27]. Where a direct dark-versus-light comparison was possible, dupilumab worked at least as well in darker skin [25]. No safety signal specific to the skin of colour was observed in any study.

That said, the confidence attached to these findings is uneven. The Black or African American subgroup was the smallest in almost every stratified trial, sometimes fewer than 100 patients, and was often reported without a formal statistical test. When a numerical difference did surface, for example, the flatter abrocitinib dose-response in Black patients, the authors attributed it to sample size rather than biology [13]. So the honest reading is not that equivalence has been proven, but that no convincing evidence of reduced benefit exists, and the darkest-skin data remain too thin to settle the question. Three separate questions should be kept apart. The first is whether each treatment worked against its comparator within a given subgroup. The second is whether the size of that effect differed between subgroups. The third is whether the treatments can be judged equivalent across groups. Efficacy shown within Black, Asian, White, or Fitzpatrick strata does not, by itself, establish equivalence among them; such equivalence would require adequately powered comparisons or formal interaction tests.

Relation to Previous Work

This review builds on and extends the synthesis by Rijal and colleagues, which is the closest prior work [9]. That review pooled AD with psoriasis, examined only biologics, and found no significant skin-of-colour differences across 11 studies, while calling for trials that stratify results by skin-of-colour status. Part of that call is addressed by this review. By keeping AD separate, adding the topical agents tapinarof, crisaborole, and roflumilast, including the JAK inhibitors, and capturing the 2025 and March 2026 stratified analyses, a fuller and more current picture of AD treatment is provided. The overall message is consistent with Rijal: treatment response does not appear to differ in skin of colour, but the evidence base is now broader and includes the first dedicated skin-of-colour trial rather than subgroup fragments alone. A search of the current literature confirmed that no existing systematic review had brought together these newer stratified and dedicated skin-of-colour datasets for AD, which supports the novelty of the present synthesis.

These results also align with the broader literature on AD in skin of colour, which has long described distinct clinical and immunological features across ethnic groups, yet has not determined whether these features alter drug response [1,2,4]. This synthesis suggests that, at least for the endpoints trials measure, they largely do not.

The Measurement Problem With Darker Skin

One theme runs under all of these results and deserves direct attention. The tools used to grade AD severity, and therefore to enrol patients and to judge response, lean heavily on erythema. Redness is harder to perceive in Fitzpatrick V and VI skin, where inflammation can appear violaceous, grey, or simply hyperpigmented, so erythema-based scoring can understate true disease activity [6]. This has two consequences for the present review. Patients with darker skin may have been scored as milder at baseline, which can shift both who entered a trial and how much room there was to improve. An efficacy result expressed through an erythema-weighted index may not capture what a patient with skin of colour actually experiences, above all, the pigmentary change that so often drives their distress. The lebrikizumab skin-of-colour trial is important, in part, because it measured pigmentation directly using an objective instrument and showed improvement in most patients with skin of colour [27]. Future trials that aim to serve this population will need outcome measures developed and validated for this population.

Clinical Implications

For day-to-day practice, the findings support a straightforward position. Clinicians should offer patients with skin of colour the same modern topical and systemic therapies, at the same thresholds, that they offer lighter-skinned patients, because the available evidence shows no clear evidence of reduced efficacy or safety. The data on pigmentation are a useful addition to counselling, since agents such as lebrikizumab and dupilumab can improve post-inflammatory dyspigmentation as the underlying disease settles [17,27]. At the same time, clinicians should be aware that standard severity scores may understate disease in their darker-skinned patients, and should weigh the patient's own report of itch, sleep, and pigmentary change alongside the erythema-based examination. Access remains a separate and real problem, since Black and Hispanic patients are less likely to receive newer agents even when eligibility is equal, but that is a question of delivery rather than of drug efficacy [7].

Strengths and Limitations

The main strength of this review is its breadth. By allowing both stratified subgroup analyses and dedicated skin-of-colour trials, and by covering every major drug class, evidence was captured that narrower, biologics-only or subgroup-only reviews would have missed, including the newest topical and dedicated-population data.

The limitations are real and mostly flow from the primary studies. Much of the efficacy evidence comes from post hoc subgroup analyses that were not powered for the subgroup, so imprecision, especially in Black or African American and Fitzpatrick V-VI patients, is a genuine threat, and several reports provided response rates without measures of dispersion or formal between-group interaction testing. Furthermore, substantial heterogeneity in drug classes, study designs, outcome measures, and reporting methods, together with insufficient quantitative data for statistical pooling, precluded a meta-analysis. Consequently, the findings were synthesised narratively rather than as a pooled effect estimate. The final study count is modest, and one key study was an open-label, single-arm trial. The search was restricted to English-language publications, which may have missed relevant data from non-English East Asian or Latin American journals. Finally, as discussed, the erythema-based endpoints that dominate the evidence may not measure disease in darker skin as well as in lighter skin, a limitation that lies within the primary data rather than in the review methodology. Restricting the search to English-language records may also have excluded Spanish-language studies of Hispanic or Latino populations, a group in which Fitzpatrick phototypes above IV are common, so some relevant data may have been missed. Because a meta-analysis was not performed, formal sensitivity analyses and statistical exploration of heterogeneity were not possible; therefore, heterogeneity was examined qualitatively, which is a further limitation of the synthesis. The Fitzpatrick skin type is also an imperfect proxy for skin colour and ethnicity, since it was designed to describe how the skin responds to sun exposure rather than a person’s race or ancestry, and grouping race, ethnicity, and phototype under a single skin-of-colour label is itself a simplification.

Future Research

The path forward is clear from the gaps. The field needs trials, or at least pre-specified and adequately powered subgroup analyses, that enrol sufficient numbers of Black, African, Hispanic, and Fitzpatrick V-VI patients to test response rather than merely describe it. It needs outcome measures validated for darker skin tones, including objective pigment endpoints, so that efficacy is judged by what matters to these patients. Dedicated skin-of-colour trials, of which ADmirable is the first, should become routine rather than exceptional. Head-to-head and longer-term data in skin of colour would help, as would work outside the East Asian and North American settings that currently supply most of the evidence, since African and South Asian populations are still barely represented.

## Conclusions

Modern topical and systemic treatments for AD are effective and well tolerated in patients with skin of colour, and the current evidence shows no clear reduction in benefit compared with that observed in lighter-skinned patients. The reassurance is real but not complete. It rests on subgroups that are often small, rarely tested formally, and weakest for the darkest skin types, and on severity tools that were not designed for melanin-rich skin. Two things would move the field from reassurance to certainty: trials that enrol and analyse enough patients with skin of colour to answer the question directly, and outcome measures that capture disease, including pigmentary change, as it truly presents in darker skin. For everyday practice, this supports offering patients with skin of colour the same modern topical and systemic therapies, at the same thresholds, as those offered to lighter-skinned patients, while looking beyond erythema-based scores and weighing the patient's own report of itch, sleep, and pigmentary change when severity and treatment response are assessed.

## Appendices

Search queries

*PubMed* 2015 to 2026 total: 435

("atopic dermatitis"[tiab] OR "atopic eczema"[tiab] OR eczema[tiab]) AND ("skin of color"[tiab] OR "skin of colour"[tiab] OR Fitzpatrick[tiab] OR "skin type"[tiab] OR "skin phototype"[tiab] OR race[tiab] OR racial[tiab] OR ethnic[tiab] OR ethnicity[tiab] OR "African American"[tiab] OR Black[tiab] OR Asian[tiab] OR Hispanic[tiab] OR Latino[tiab]) AND (treatment[tiab] OR therapy[tiab] OR efficacy[tiab] OR effectiveness[tiab] OR response[tiab])

*Scopus* 2015 to 2026 total: 1390 TITLE-ABS-KEY("atopic dermatitis" OR "atopic eczema" OR eczema) AND TITLE-ABS-KEY("skin of color" OR "skin of colour" OR Fitzpatrick OR "skin type" OR "skin phototype" OR race OR racial OR ethnic OR ethnicity OR "African American" OR Black OR Asian OR Hispanic OR Latino) AND TITLE-ABS-KEY(treatment OR therapy OR efficacy OR effectiveness OR response)
